# The Timing of Application and Inclusion of a Surfactant Are Important for Absorption and Translocation of Foliar Phosphoric Acid by Wheat Leaves

**DOI:** 10.3389/fpls.2019.01532

**Published:** 2019-11-22

**Authors:** Courtney A. E. Peirce, Therese M. McBeath, Craig Priest, Michael J. McLaughlin

**Affiliations:** ^1^Soil Science, School of Agriculture, Food and Wine, The University of Adelaide, Glen Osmond, SA, Australia; ^2^CSIRO Agriculture and Food-Systems, Glen Osmond, SA, Australia; ^3^Future Industries Institute, University of South Australia, Mawson Lakes, SA, Australia

**Keywords:** foliar uptake, phosphorus, adjuvant, surfactant, wettability

## Abstract

**Introduction:** Foliar applied phosphorus (P) has the potential to provide a more tactical approach to P fertilization that could enhance P use efficiency. The aims of this study were to investigate the influence of adjuvant choice and application timing of foliar applied phosphoric acid on leaf wettability, foliar uptake, translocation, and grain yield of wheat plants.

**Materials and Methods:** We measured the contact angles of water and fertilizers on wheat leaves, and the uptake, translocation and wheat yield response to isotopically-labelled phosphoric acid in combination with five different adjuvants when foliar-applied to wheat at either early tillering or flag leaf emergence.

**Results:** There was high foliar uptake of phosphoric acid in combination with all adjuvants that contained a surfactant, but only one treatment resulted in a 12% increase in grain yield and two treatments resulted in a decrease in grain yield. Despite the wettability of all foliar fertilizers being markedly different, foliar uptake was similar for all treatments that contained a surfactant. The translocation of phosphorus from foliar sources was higher when applied at a later growth stage than when applied at tillering despite the leaf surface properties that affect wettability being similar across all leaves at both growth stages.

**Discussion:** Both the timing of foliar application and the inclusion of a surfactant in the formulation are important for absorption and translocation of phosphoric acid by wheat leaves, however high foliar uptake and translocation will not always translate to a yield increase.

## Introduction

Phosphorus (P) is an essential nutrient needed for plant growth, but due to chemical reactions in soil, it has low immediate availability and limited efficiency in the year of fertilizer application. Fertilizer P requirement of crops in Mediterranean cropping systems is highly dependent on seasonal rainfall. As a result, fertilizer can be a high-risk input cost for farmers, especially in areas of southern Australia with variable rainfall (Kingwell, 2011). Current best practice is to apply all P fertilizer at sowing, banded below the seed, when predicting rainfall driven yield potential can be unreliable (McLaughlin et al., 2011). However, applying all P fertilizer at sowing, when the season ahead is unknown, increases the risk associated with the fertilizer investment. Hence the potential to use an in-season foliar P top-up as a tactical management technique in response to favourable seasonal conditions is attractive. A tactical management approach has been used for nitrogen (N) to increase grain protein content, but has been inconsistent at increasing grain yields (Gooding and Davies, 1992; Woolfolk et al., 2002; Bly and Woodard, 2003; Varga and Svecnjak, 2006). Foliar application with micronutrients has been used extensively especially in horticulture to alleviate nutrient deficiencies and maintain plant health (Fernández and Eichert, 2009), but use with macronutrients both in horticulture and cropping systems has been limited.

In order for foliar fertilization to be successful the nutrients must be able to penetrate the outer protective layer of the leaf and absorbed through the cuticle, cuticular irregularities, stomata, trichomes, or other epidermal structures to reach the internal cells of the plant (Fernández and Eichert, 2009; Fernández and Brown, 2013). To then be beneficial to the plant, there must be movement from the site of application to other plant cells. One benefit of P in this respect is that it is a mobile element in the phloem and is readily transported and redistributed around the plant, unlike other nutrients including calcium and boron which are relatively phloem immobile (White, 2012). It has also been shown that the P can be translocated not only in the phloem but also the xylem (Martin, 1982), and as a result at maturity, depending on the P status of the plant, translocation of P from other plant parts can account for 20 to 90% of the P in the grain (Batten and Wardlaw, 1987; Peng and Li, 2005). However, before both uptake and translocation can occur, the foliar fertilizer must adhere to the leaf.

The wettability of plant leaves as well as the ability of leaves to absorb and translocate foliar-applied fertilizers can vary with individual leaf age and crop growth stage (Sargent and Blackman, 1962; Troughton and Hall, 1967; Puente and Baur, 2011). In addition to the implication this has for uptake of foliar fertilizers, there must also be nutrient demand at the growth stage corresponding to when the spray is applied for the application to be beneficial. The maximum possible interception of foliar sprays is controlled by the crop cover and area of leaf available to intercept the spray. For foliar applications of P, this is essential because of the limited mobility of P in soil, particularly if the P is surface applied where the soil can dry out, thus further limiting P movement (Marschner and Rengel, 2012). The supply of P is critical during early plant growth with wheat yields substantially reduced if P supply is limited (Grant et al., 2001). For maximum grain yield to be achieved, P uptake is required until heading (Boatwright and Viets, 1966) or anthesis (Batten et al., 1986) with supply post-anthesis suggested to have no effect on grain yields. This is despite substantial soil P uptake occurring after anthesis in some studies (Mohamed and Marshall, 1979). Crop P uptake from soil accumulates rapidly between stem elongation and anthesis which also coincides with the period of maximum leaf area (Waldren and Flowerday, 1979). Past studies have suggested the optimal timing of foliar P application is from canopy closure to anthesis (reviewed in Noack et al., 2011).

The adhesion of foliar fertilizers is particularly important for wheat leaves compared to some other broadacre crops. This is because wheat plants have been shown to have leaves that are particularly difficult to wet due to the surface roughness induced by extra cuticular waxes and leaf hairs (trichomes) (Holloway, 1969; Netting and von Wettstein-Knowles, 1973; de Ruiter et al., 1990). The surface roughness can decrease wettability where the contact angle of water on the leaf surface can be as high as 160 degrees, with little resultant adhesion of water (Fernández et al., 2014). It has been suggested that improved wetting of foliar fertilizer on leaves (i.e., a small contact angle measured at the leaf-fertilizer interface), will increase the chance of uptake for foliar-applied fertilizers (Fernández and Eichert, 2009). It follows that poorer wetting (higher contact angles) will result in lower uptake rates of the fertilizer formulation.

To improve the efficacy of foliar uptake, the addition of adjuvants to the fertilizer formulation is often required (Fernandez and Eichert, 2009). Adjuvants are defined as any material that is added to a spray solution to enhance uptake of the active ingredient of the spray, whether the spray be a fertilizer, herbicide or pesticide, or to modify the spray characteristics (Hazen, 2000). Adjuvants can include oils, pH buffers, surfactants, humectants, or mixtures which can contain multiple modes of action (Somervaille et al., 2014). The addition to sprays of oils, which can act as a penetrating agent, and the addition of humectants and surfactants are common for lipophilic herbicides and pesticides, which are often sparingly soluble in water (Hazen, 2000; Somervaille et al., 2014). The use of pH buffers is also important for herbicides due to greater effectiveness of many active ingredients at a low pH compared to higher pH (Somervaille et al., 2014). Of more relevance to foliar fertilizers is the use of surfactants, which work by lowering the surface tension of the formulation to improve spreading and adhesion of the fertilizer on the leaf surface and therefore, increase the leaf area in contact with the fertilizer (Fernández and Eichert, 2009). In addition to their effect on the wettability of the adaxial surface (Januszkiewicz et al., 2019), there is also some evidence that surfactants penetrate the plant cuticle or increase the water conductance of the cuticle and as a result increase the rate of foliar uptake (Hess and Foy, 2000; Räsch et al., 2018). Humectants may also be useful in improving uptake of foliar fertilizers. This is because humectants increase the drying time of aqueous sprays (Hazen, 2000), which is essential as foliar uptake only occurs when the fertilizer is in a liquid form (Fernández and Eichert, 2009). There are a large number of adjuvants which are commercially available for use in combination with agrochemicals, however many provide no label recommendations for use with foliar-applied fertilizers (Somervaille et al., 2014). There is potential for incompatibility between P-containing components and adjuvants in the formulation.

To measure the effect of adjuvants on uptake and translocation of foliar-applied phosphoric acid, two commercial adjuvant mixtures, two laboratory grade surfactants, and one laboratory grade humectant were applied in combination with ^33^P-labelled phosphoric acid at two different growth stages to wheat plants grown under controlled conditions. These two growth stages were chosen to represent a time of high P requirement by the plant (early tillering) and towards the end of peak P demand when there is more ground cover and opportunity for interception (flag leaf emergence). This study aimed to investigate whether the choice of adjuvant influences the uptake and distribution of foliar-applied P when plants were grown through to maturity and also, whether any of the foliar formulations resulted in an increase in wheat yield when grown in a soil with marginal soil P availability.

## Materials and Methods

### Soil Collection and Chemical Properties

The soil used in this study was classified as a Calcisol according to the World Reference Base (FAO, 2006) and was collected from an agricultural site near Black Point on the Yorke Peninsula in South Australia (S34°36.776’, E137°48.599). It has a sandy loam A horizon which overlies calcareous substrate. Soil characteristics have been described in Peirce et al. (2014). Briefly, the soil is a loam with a pH (1:5 in H_2_O) of 8.5 that has no detectable calcium carbonate or surface salinity issues. The cation exchange capacity (CEC) is 17.9 cmol kg^-1^ and it has an organic carbon content of 1.6 g kg^-1^. The soil is classified as P-deficient with plants grown in the soil likely to be P-responsive (measured Colwell-P 3 mg kg^-1^, PBI 75 and DGT-P 4 µg L^-1^) (McBeath et al., 2007).

### Growth Conditions

Plants were grown in pots of 15 cm diameter and 17 cm depth that were not free-draining and held a total of 3 kg soil pot^-1^. Before sowing, the soil was wetted to 15% of field capacity (Klute, 1986) and the following basal nutrients were mixed through the soil: potassium (K) as K_2_SO_4_ at 200 mg K pot^-1^ (113 kg K ha^-1^), magnesium (Mg) as MgSO_4_.7H_2_O at 50 mg Mg pot^-1^ (28 kg Mg ha^-1^), zinc (Zn) as ZnSO_4_.7H_2_O at 30 mg Zn pot^-1^ (17 kg Zn ha^-1^), copper (Cu) as CuSO_4_.5H_2_O at 24 mg Cu pot^-1^ (14 kg Cu ha^-1^), manganese (Mn) as MnCl_2_ at 4 mg Mn pot^-1^ (2 kg Mn ha^-1^), and the total sulfur (S) applied in these reagents equated to 175 mg S pot^-1^ (57 kg S ha^-1^). The soil was watered to 70% field capacity and P and N were added to the soil as a band 2 cm beneath the seed at a rate of 12 mg P pot^-1^ (6.6 kg P ha^-1^) as phosphoric acid and 150 mg N pot^-1^ (85 kg N ha^-1^) as urea before sowing. At early tillering, 18 days after sowing (DAS) additional 75 mg N pot^-1^ as urea and 7.5 mg of Zn as ZnSO_4_.7H_2_O were applied in solution to the soil surface and watered in.

Four seeds of wheat (*Triticum aestivum* L. cv. Axe) that had been germinated a few days prior were sown in each pot at 10 mm depth and thinned at the two-leaf growth stage by leaving the two most uniform seedlings per pot. The cultivar Axe is a fast-developing Spring wheat and was chosen to reduce the radiation load required for the fertilizer labelling with ^33^P. Immediately after sowing, the surface of the pot was covered with 80 g of alkathene granules to minimise evaporation from the soil. Pots were watered every two days to maintain 80% field capacity before increasing watering frequency to every day from early booting. Plants were grown in a controlled environment room (20°C/15°C day/night cycle of 12 h each) and their positions on benches randomised every week. The plants were moved to the glasshouse 67 DAS to ripen and watering was suspended 89 DAS, two weeks before harvest. Average growing conditions in the glass house were 23.2°C with 60.9% relative humidity (RH) (minimum 15.9°C 13.7% RH and maximum 35.8°C 92.5% RH).

### Adjuvants

We used five different adjuvants including two commercial products, two pure surfactants, and a humectant, namely: LI 700^®^, Agral^®^, Genapol^®^ X-080, Triton™ X-100, and glycerol. LI 700^®^ is an acidifying and penetrating mixture with 35% w v^-1^ propionic acid (CAS No. 79-09-4), 35% w v^-1^ soyal phospholipids (CAS No. 8002-43-5), and 10–30% w v^-1^ non-ionic surfactant. It also acts as a pH buffer by acidifying the spray solution. Agral^®^ is a spray additive with 63% w v^-1^ nonyl phenol ethylene oxide condensate (non-ionic surfactant) (CAS No. 9016-45-9). Genapol^®^ X-080 is a pure non-ionic surfactant of polyethylene glycol monoalkyl ether (CAS No. 9043-30-5). Glycerol is a simple polyol (CAS No. 56-81-5), which is hygroscopic and therefore acts as a humectant. Triton™ X-100 is a non-ionic surfactant of p-tertiary-octophenoxy polyethyl alcohol (CAS No. 9002-93-1).

### Foliar Application

Foliar treatments consisted of five adjuvants (one concentration each) at two different foliar application timings to give 10 treatments with five replicates for each treatment. A further five replicates were included without foliar P application as a control and another five replicates for destructive measurements (contact angles and scanning electron microscopy). This gave a total of 60 pots.

The two foliar applications were 21 DAS corresponding to plants at early tillering (Zadoks GS21) and 32 DAS corresponding to plants with the flag leaf collar visible [Zadoks 39 (Zadoks et al., 1974)]. The GS21 timing was applied to the largest fully expanded leaf tiller, and the second (L2) and third leaf (L3) counting up from the base of the main stem (MS). For the second application timing (GS39), another leaf from the main stem corresponding to the penultimate main stem leaf (i.e. leaf below the flag leaf), L4, was also treated. The rate of foliar applied P for all treatments was 3.4 mg of P pot^-1^ as phosphoric acid, equivalent to approximately 1.9 kg P ha^-1^ at a watering rate of 100 L ha^-1^ (based on pot surface area). The concentration of adjuvant used depended on the adjuvant in question but was the label rate for Agral^®^ and LI 700^®^ (1 and 3 g L^-1^ respectively) and 1 g L^-1^ for the other three adjuvants. Each foliar fertilizer was labelled with carrier-free ^33^P in the orthophosphate form to give a spike rate of 0.5 MBq pot^-1^ at application. Before foliar P application, the soil surface was covered with plastic wrap to ensure any drops that did not adhere to the leaves would not reach the soil surface. The foliar fertilizers were applied mid-morning to the five most prominent fully expanded leaves for each plant at the time of application. This corresponded to two leaves on the tillers and three on the main stem at both timings to give ten leaves per pot. Drops were applied with a micropipette to the adaxial leaf side totalling 177 µl pot^-1^ split between all the leaves. Drop size was consistent between all the treatments (4-5 µl) except glycerol which, due to the difficulty in detaching the droplets from the micropipette, were much larger (average of 12 µl and 10 µl for the two timings respectively). The estimated loss of foliar fertilizer through droplet movement (non-adherence to the leaf) was recorded by visual observation of each deposited drop. All treated leaves were marked for easy identification and three days after application each treated leaf was scored for leaf burn according to a modification of the method of Stein and Storey (1986), namely 1 = no effect, 2 = slight surface burn on the treated area without apparent cell collapse, 3 = slight to heavy burn on the treated area only with some cell collapse, 4 = heavy surface burn extending between treated areas, 4.5 = the same as 4 with leaf tip senescence, and 5 = leaf dead.

### Leaf Surface Characteristics

Wheat leaves corresponding to the treated leaves from the foliar application experiment were collected at both early tillering (Zadoks 21) and when the flag leaf collar was visible/late flag leaf emergence [Zadoks 39 (Zadoks et al., 1974)] and small sections from the middle of the leaf (avoiding the mid-rib) were cut and fixed for scanning electron microscopy (SEM). The leaf samples were fixed and vacuum infiltrated in 0.25% gluteraldehyde, 4.0% paraformaldehyde in phosphate buffer solution (PBS) with 4% sucrose at a pH of 7.2 overnight. Samples were then rinsed in PBS and 4% sucrose three times before post fixing in 2% osmium tetroxide in PBS for 1 h. They were then washed and progressively dehydrated in an ethanol series: 70 (two changes of 15 min), 90 (two changes of 15 min), and 100% ethanol (three changes of 15 min). Samples were then critical point dried in a Bal-tec CPD 030 Critical Point Dryer, mounted on a stub and coated with a 5 nm layer of platinum. Images were taken on a Philips XL20 scanning electron microscope under high vacuum at 10 kV and a working distance of 10 mm. Stomatal and trichome densities were calculated by analysing images taken by SEM.

### Contact Angle Measurements on Leaves

The static advancing and receding contact angle of water and fertilizers was measured on the adaxial (upper) side of leaves from wheat plants at growth stages corresponding to the two foliar application timings [Zadoks 21 and Zadoks 39, (Zadoks et al., 1974)]. Measurements were made using the sessile drop method (with the needle in) and calculated based on observation of the profile of small water droplets (1-2 µl) (DataPhysics, OCAH 200) as described in Forsberg et al. (2010). The initial droplet volume was brought into contact with the surface, increased slowly until the contact line advanced, and then stopped before measurement. In the same way the liquid volume of the drop deposited onto the surface was decreased until the contact angle receded, and then was stopped before measurement. All contact angle measurements were made on the mid-section (length-wise) of the leaf between the leaf edge and mid-vein. To do this, sections of the leaf were cut and stuck to glass slides with double-sided tape. Care was taken to avoid damage to the leaf surface: the leaf was only handled on its edges away from where contact angle measurements were made. Unlike water, adjuvant drops were allowed to relax for 20 s before contact angle measurements were taken. This was due to the dynamics of the adjuvants at the leaf surface causing the drop to spread over time rather than remain a static contact angle. A time of 20 s was chosen to allow a more reproducible angle to be measured (Peirce et al., 2016). A final contact angle was not measured but has been shown for three of the five adjuvants to effectively reach zero if allowed enough time to spread (Peirce et al., 2016). For fertilizers on leaves, the receding contact angle could not be measured as the droplet was not observed to recede from the leaf surface, i.e. the receding contact angle was effectively 0° in every case except for glycerol. Contact angle values reported for fertilizers are the average of 12 measurements taken over three leaves (corresponding to those treated in the plant experiment) and contact angle values for water are the average of 15–25 measurements taken over all leaf sections analysed.

### Foliar Uptake and Translocation

Above-ground plant parts were harvested at maturity when grains were ripe [Zadoks 92, (Zadoks et al., 1974)]. Plant parts were harvested 1 cm from above the soil surface and divided into the following sections before washing: treated leaves; untreated leaves; heads; and stems. Each of these plant parts was washed for 30 s in 100 ml of 0.05% w v^-1^ Triton^™^ X-100 + 0.1 M HCl then rinsed in RO water for 20 s and DI water for 20 s (Fernández et al., 2014). The first washing solution was kept for analysis of total P by inductively coupled plasma-atomic emission spectroscopy (ICP-AES) and ^33^P activity on the beta counter. All parts were dried in an oven at 60°C for 72 h. Plant parts were weighed and the grain was separated from the chaff. All plant parts were digested in boiling nitric acid and analysed for P by ICP-AES (Zarcinas et al., 1987). A 2 ml sample of the digest was added to a vial with 10 ml of scintillation fluid (EcoScint) and counted on a Perkin Elmer Quanta Smart liquid scintillation analyser (Model Tri-Carb B3110TR). All counts were blank corrected and corrected for decay to a single time point (harvest).

### Calculations and Statistical Analysis

The amount of foliar P absorbed (P uptake) was expressed as a percentage and calculated as the amount of ^33^P recovered in washed plant parts divided by the ^33^P in the applied fertilizer. The translocation was also expressed as a percentage of the total ^33^P in the applied fertilizer and consisted of the ^33^P recovered in all washed plant parts minus the treated leaves divided by the ^33^P in the applied fertilizer.

(1) $$P\;uptake\;\left(\%\right)\;=\;\frac{\;33P_{in\;plant\;parts\;from\;fertilizer}\left(\frac{mg\;P}{pot}\right)}{\;33P_{in\;foliar\;fertilizer}\;\left(\frac{mg\;P}{pot}\right)}\;\times 100$$

(2) $$\begin{array}{l} Translocation\;\left(\%\right)\;=\;\\ \;\;\;\;\;\;\frac{\;P_{in\;plant\;parts\;from\;fertilizer}\left(\frac{mg\;P}{pot}\right)-\;P_{in\;treated\;leaves}\left(\frac{mg\;P}{pot}\right)}{\;P_{\;in\;foliar\;fertilizer}\;\left(\frac{mg\;P}{pot}\right)}\;\times 100 \end{array}$$

The plant P derived from the foliar fertiliser was simply the ^33^P radioactivity of the washed plant parts divided by the specific activity (SA) of the foliar fertiliser.

P derived from foliar fertilizer​ = (mgpot)P derived from foliar fertilizer​ = 33Pradioactivity in plant parts(Bqpot)SAof foliar fertilizer (Bqmg P)

Statistical analysis was performed by analysis of variance (ANOVA) in the Genstat^®^ V.15 statistical package. Both the normality of distribution and constant error variance assumptions were tested for each analysis. Differences between treatments were determined by least significant difference (l.s.d.) at the 5% significance level using Fisher’s protected l.s.d. The treatment structure run in ANOVA for all analysis that included controls (dry weight and P uptake) was foliar/(timing × adjuvant) at 2 levels = yes (all timing x adjuvant combinations) or no (controls); timing at 2 levels = early tillering or flag leaf emergence and adjuvant at 5 levels = Glycerol, Agral^®^, LI 700^®^, Triton™ X-100 or Genapol^®^ X-080. The treatment structure for all other analysis undertaken in ANOVA was adjuvant × timing.

## Results

### Plant Growth

The foliar application of phosphoric acid with LI 700^®^ at flag leaf emergence produced the only positive grain yield response of 12% more grain than the control (**Table 1**). Conversely, when the LI 700^®^ treatment was applied at early tillering, it produced 22% less grain than the control. The foliar application of phosphoric acid in combination with Genapol^®^ X-080 resulted in a decrease in grain yield of 12% when applied at early tillering. There were no differences between treatments in total above-ground plant biomass or stem biomass at harvest (**Table 1**). Only the LI 700^®^ treatment applied at early tillering had lower leaf and chaff biomass than the control, corresponding with the reduction in grain yield. There was also a significant effect of timing of application for the weight of stems, leaves, and whole plants. Plants fertilised at early tillering had lower stem and whole plant biomass than the control. Foliar application at flag leaf emergence did not result in any differences in stem, leaf, or whole plant biomass compared to the control plants. Neither 1,000-grain weight nor grain number (grand mean of 157 pot^-1^) showed any differences between treatments (**Table 2**). There were also no differences in the P content or P concentration of the grain between any of the treatments (**Table 2**).

**Table 1:** Effect of foliar treatments on shoot dry weight.

	Grain	Chaff	Stems	Leaves	Whole plant
	*(g pot* *^-1^* *)*				
***Foliar.Adjuvant.Timing***					
Control (no foliar)	5.24 ^bc^	1.94 ^ab^	2.23	1.79 ^abc^	11.20
***Early tillering (Z21)***					
Glycerol	5.55 ^abc^	1.98 ^ab^	2.07	1.79 ^abc^	11.40
LI 700^®^	4.09 ^e^	1.43 ^c^	1.48	1.34 ^d^	8.34
Triton™ X-100	5.10 ^cd^	1.86 ^ab^	1.93	1.67 ^bcd^	10.56
Agral^®^	5.66 ^ab^	1.94 ^ab^	1.90	1.76 ^abc^	11.27
Genapol^®^ X-080	4.63 ^de^	1.76 ^bc^	1.79	1.58 ^cd^	9.77
***Flag leaf emergence (Z39)***					
Glycerol	5.53 ^abc^	2.05 ^ab^	2.47	1.97 ^ab^	12.03
LI 700^®^	5.88 ^a^	2.21 ^a^	2.28	2.08 ^a^	12.46
Triton™ X-100	5.32 ^bc^	1.89 ^ab^	2.18	1.91 ^abc^	11.29
Agral^®^	5.19 ^bc^	1.80 ^abc^	1.95	1.79 ^abc^	10.74
Genapol^®^ X-080	5.52 ^abc^	1.97 ^ab^	1.94	1.87 ^abc^	11.31
*LSD (p ≤ 0.05)*	*0.54*	*0.43*	*n.s.*	*0.33*	*n.s.*
***Foliar.Timing***					
No foliar application	5.24	1.94	2.23 ^a^	1.79 ^ab^	11.20 ^a^
Early tillering *(Z21)*	5.01	1.79	1.84 ^b^	1.63 ^b^	10.27 ^b^
Flag leaf emergence *(Z39)*	5.49	1.98	2.17 ^ab^	1.93 ^a^	11.57 ^a^
*LSD (p ≤ 0.05)*	*n.s.*	*n.s.*	*0.35*	*0.26*	*0.86*

Statistical differences within a column and treatment design indicated with different letters (p ≤ 0.05, LSD in table).

n.s. indicates not significant.

**Table 2:** Effect of foliar treatments on grain number, P content and P concentration.

	Grain number	Grain P content	Grain P concentration
	*grains pot* *^-1^*	*mg pot* *^-1^*	*mg kg* *^-1^*
***Foliar.Adjuvant.Timing***			
Control (no foliar)	159	12.5	2400
***Early tillering (Z21)***			
Glycerol	162	14.4	2594
LI 700^®^	125	11.4	2803
Triton™ X-100	156	14.4	2838
Agral^®^	176	17.1	3011
Genapol^®^ X-080	140	13.1	2850
***Flag leaf emergence (Z39)***			
Glycerol	166	14.4	2619
LI 700^®^	174	15.9	2654
Triton™ X-100	155	14.1	2717
Agral^®^	152	14.8	2862
Genapol^®^ X-080	163	14.7	2660
*LSD (p ≤ 0.05)*	*n.s.*	*n.s.*	*n.s.*
**Grand Mean**	**157**	**14.3**	**2728**

Statistical differences within a column indicated with different letters (p ≤ 0.05, LSD in table).

n.s. indicates not significant.

**Figure 1:**
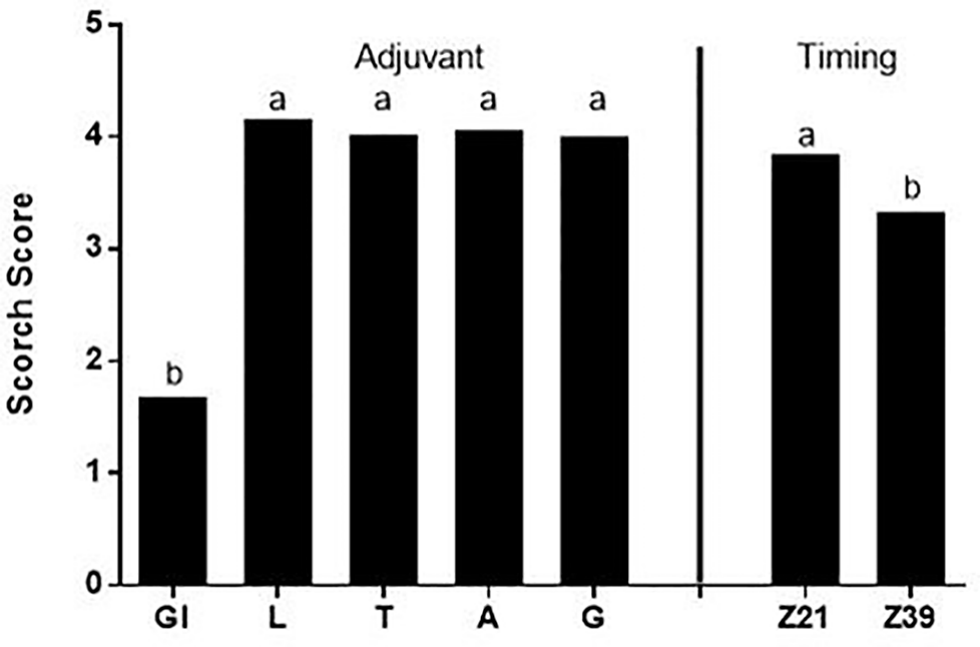
Average scorch score for adjuvants and timing; there was no significant adjuvant by timing interaction, Treatments: Gl-Glycerol, L-LI 700^®^, T-Triton™ X-100, A-Agral^®^ and G- Genapol^®^ X-080. Statistical differences between average scorch score for adjuvant (p ≤ 0.05, l.s.d. 0.27) and timing (p ≤ 0.05, l.s.d. 0.17) indicated on graph with different letters.

There was no relationship between the scorch score and either the above ground dry weight or grain weight (data not shown). The scorch score for all treatments except glycerol was high representing visible necrosis and senescing of at least some leaves within each pot when scorch was measured three days after foliar application (**Figure 1**). There was both an adjuvant effect due to the lower scorch from glycerol treatments and a timing effect with application of foliar fertilizer at the later timing resulting in less severe scorch.

### Plant Surface and Contact Angles

**Figure 2:**
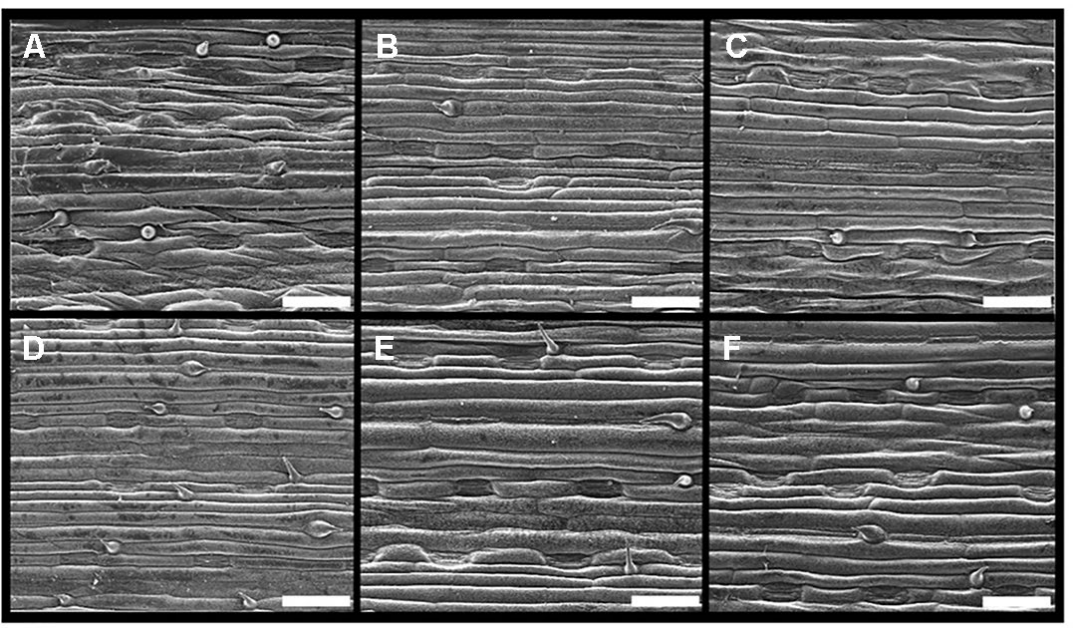
Scanning electron microscope images of the adaxial side of wheat leaves: **(A**–**C)** at early tillering Z21, **(D**–**F)** and at flag leaf emergence Z39. **(A** and **D)** leaf on first tiller, **(B** and **E)** Leaf 2 from main stem base, **(C** and **F)** Leaf 3 from main stem base; scale bar = 100 µm.

**Figure 2** shows the adaxial leaf side of wheat leaves taken by scanning electron microscope corresponding to the growth stages at which foliar P was applied. The leaves shown correspond to leaves that had foliar fertilizers applied to them Although there appears to be slightly different densities of stomata and trichomes ranging from 42–65 stomata mm^-2^ and 13–42 trichomes mm^-2^ across the treated leaves (**Table 3**), this is likely to be natural variation as the wettability (measured by advancing and receding contact angles of water) was not significantly different between the leaves or the timings (grand mean of 162° and 154° for advancing and receding contact angles respectively).

**Table 3:** Number of stomata and trichomes mm^-2^ ± standard deviation on the adaxial side of leaves representative of foliar-treated leaves (counted using scanning electron microscopy).

Timing	Leaf	Stomata	Trichomes
		*No. mm* *^-2^*	
***Early tillering (Z21)***	L2	56 ± 12	16 ± 4
	L3	46 ± 7	21 ± 8
	tiller	49 ± 7	37 ± 4
***Flag leaf emergence (Z39)***	L2	42 ± 12	13 ± 4
	L3	59 ± 4	20 ± 7
	L4	65 ± 7	42 ± 6
	tiller	56 ± 9	37 ± 15
**Average both timings**		**55 ± 12**	**27 ± 13**

Leaf number (L2 etc.) corresponds to the leaf count from the base of the main stem upwards.

**Figure 3:**
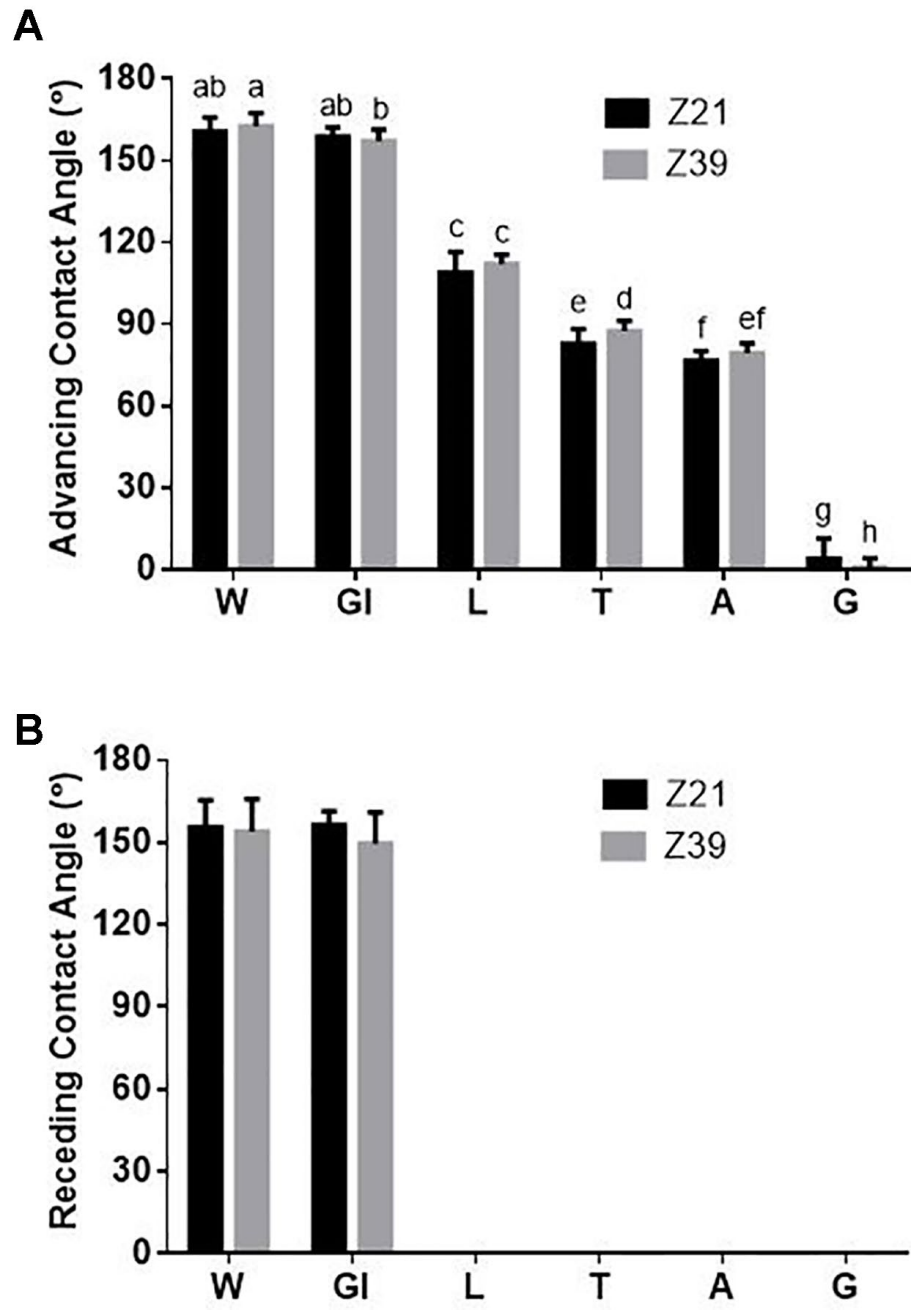
Average **(A)** advancing and **(B)** receding contact angle on adaxial side of fully expanded wheat leaves (tiller and main stem leaves) at 20 s for water and each of the adjuvants at both foliar timings (+/- standard deviation), Treatments: W-water, Gl-Glycerol, L-LI 700^®^, T-Triton™ X-100, A-Agral^®^ and G- Genapol^®^ X-080. Statistical differences between advancing contact angles indicated on graph with different letters (p ≤ 0.05, l.s.d. 3.95).

From the measurement of contact angles of the fertilizer treatments on leaves we found that there was not a timing effect but there was a formulation treatment effect (**Figure 3**). Contact angles measured 20 s after the formulation touched the leaf surface showed that, except for glycerol, all adjuvant treatments significantly decreased the advancing contact angle of the drop, but to different degrees depending on the adjuvant ranging from 111° for LI 700^®^ to 0° for Genapol^®^ X-080 (**Figure 3A**). When fertilizer drops of phosphoric acid with glycerol were deposited on the growing leaves, more drops did not adhere when applied at early tillering compared to flag leaf emergence (**Table 4**; estimated run-off). The receding contact angle for all these treatments (except glycerol) was also effectively zero as the drop could not be removed from the leaf once it was deposited (**Figure 3B**). All these adjuvants also had a spreading dynamic, continuing to spread on the leaf surface until the drop dried out. For glycerol however, both advancing and receding contact angles were similar to water although slightly lower than water when applied at flag leaf emergence (advancing 158° ± 4, receding 153° ± 9).

**Table 4:** Foliar fertiliser recovery in the plant, washing solution and run-off from different foliar treatments.

	Plant P uptake	P in wash	Run-off (estimated)	Residual
	*Phosphorus (as a % of foliar fertiliser P applied)*			
***Adjuvant.Timing***				
***Early tillering (Z21)***				
Glycerol	7.8 ^d^	0.3 ^f^	80.1 ^a^	11.9
LI 700^®^	81.0 ^ab^	3.0 ^b^	0.5 ^c^	15.4
Triton™ X-100	82.4 ^ab^	4.5 ^a^	0.5 ^c^	12.5
Agral^®^	81.6 ^ab^	2.7 ^bc^	1.8 ^c^	12.2
Genapol^®^ X-080	82.1 ^ab^	3.0 ^b^	0.0 ^c^	14.9
***Flag leaf emergence (Z39)***				
Glycerol	27.4 ^c^	0.9 ^ef^	61.8 ^b^	9.8
LI 700^®^	71.4 ^b^	1.2 ^def^	0.4 ^c^	27.0
Triton™ X-100	83.5 ^a^	1.9 ^cde^	0.5 ^c^	14.1
Agral^®^	79.8 ^ab^	2.1 ^bcd^	1.8 ^c^	16.4
Genapol^®^ X-080	74.9 ^ab^	2.7 ^bc^	0.9 ^c^	21.5
*LSD (p ≤ 0.05)*	*11.7*	*1.03*	*5.2*	*n.s.*

Statistical differences within a column indicated with different letters (p ≤ 0.05, LSD in table). *Estimated run-off loss was calculated based on visual observation of number of drops that did not adhere to the leaf.*

### Plant P Uptake and Translocation

**Figure 4:**
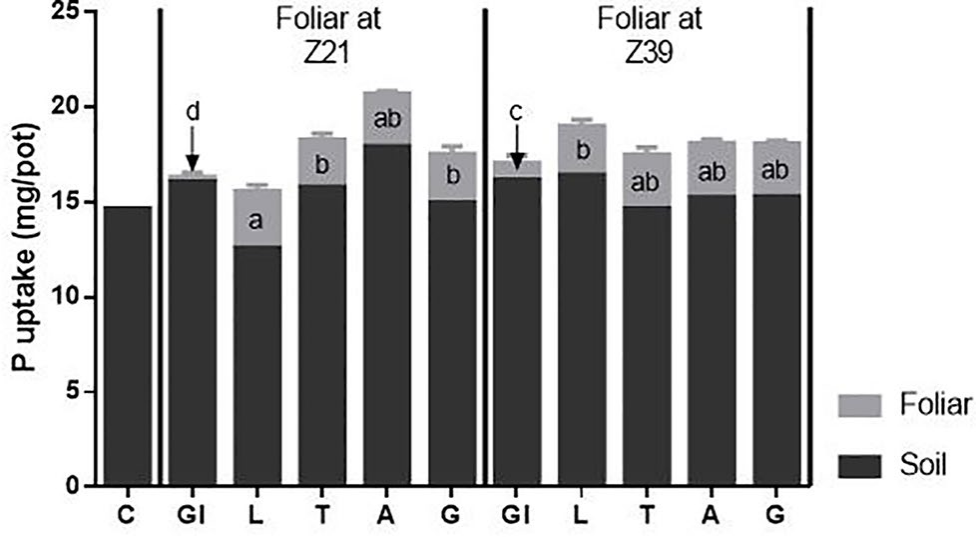
Source of P taken up by above-ground plant parts. Treatments: C-control, Gl-Glycerol, L-LI 700^®^, T-Triton™ X-100, A-Agral and G- Genapol**^®^** X-080. Statistical differences between foliar P treatments (at both times) indicated on graph with different letters (p ≤ 0.05 l.s.d 0.37).

Despite foliar treated plants receiving additional P in the foliar fertilizer, the total P uptake of the plants and P derived from the soil was not different from control plants (**Figure 4**). Importantly, there were differences in the uptake of P derived from the foliar source between foliar treatments. At both timings, plants from the glycerol treatment had significantly less foliar P than from all the other foliar treatments. Timing also proved important with less P derived from the foliar application when applied at early tillering compared to flag leaf emergence. Plants with the foliar LI 700^®^ treatment applied at early tillering had more P derived from the foliar application than when applied at flag leaf emergence.

The uptake of foliar P as a percentage of P applied was similar for all adjuvant treatments across both timings (averaging 79.6%) except for glycerol treatments, which were considerably lower (**Table 4**). For glycerol treatments, there was higher uptake at the second timing (27.4 compared to 7.8%) due to higher drop adhesion (lower estimated loss due to run-off; **Table 4**) suggesting that leaves were more wettable at the second timing despite the contact angle data not showing differences between timings (**Figure 3**). In all cases, only a small percentage of the foliar fertilizer P that adhered to the leaves was washed off (less than 5%), with the smallest percentage from glycerol treatments (**Table 4**). Any fertilizer not recovered as plant uptake, in the washings or estimated as run-off loss was classified as unrecovered foliar fertilizer P. Although there were no differences between treatments, this accounted for 10–27% of the foliar P applied.

**Figure 5:**
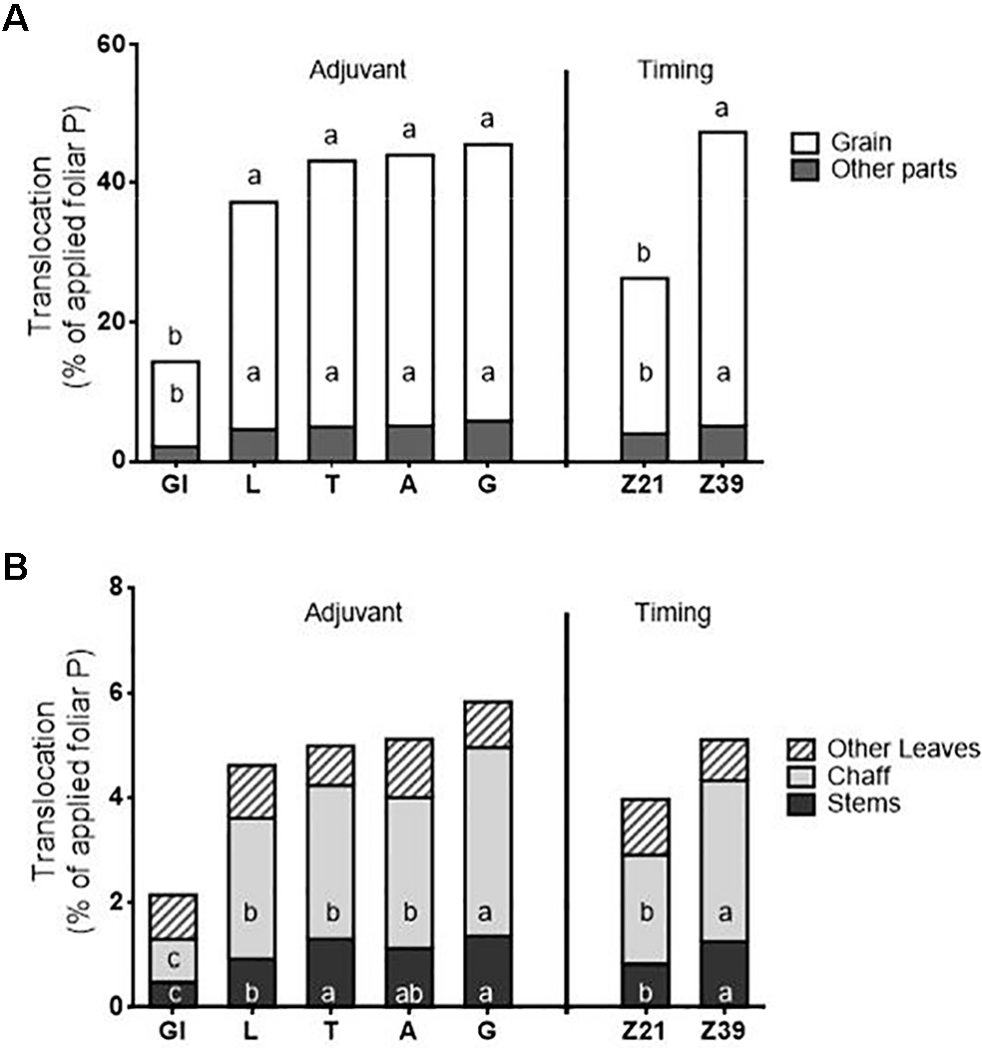
Translocation of foliar P to above-ground plant parts as a percentage of applied fertiliser; **(A)** total translocation and translocation to grain vs. the other plant parts, **(B)** expansion of translocation to other plant parts/Treatments: C-control, Gl-Glycerol, L- LI 700^®^, T-Triton™ X-100, A-Agral^®^ and G- Genapol^®^ X-080. Statistical differences within a factor and plant part for foliar P translocation indicated on graph with different letters (p ≤ 0.05; for adjuvant: total translocation l.s.d. 6.0, grain l.s.d. 5.2, other leaves n.s., chaff l.s.d. 0.6, stems l.s.d. 0.3; for timing: total translocation l.s.d. 3.8, grain l.s.d. 3.3, other leaves n.s., chaff l.s.d. 0.4, stems l.s.d. 0.2)

There was both an adjuvant and timing effect, but not an interaction for foliar translocation of P expressed as a percentage of applied foliar P (**Figure 5**). Due to the reduced uptake of P in the glycerol treatment (due to fertilizer not adhering to the leaf), glycerol-treated plants also had lower total translocation, and translocation to the grain, chaff, and stem from the foliar treated area than the other adjuvant treatments. There were no differences in either total translocation (averaging 43%), or translocation to individual plant parts between the other four adjuvants (which all contained surfactants). The total translocation and translocation to all plant parts except the leaves was also higher when applied at flag leaf emergence than at tillering. For all treatments regardless of adjuvant used or timing, the largest sink for translocated P was the grain.

## Discussion

The timing of application appears to be more important than the adjuvant choice with early application resulting in leaf damage, which reduced the plant’s ability to translocate nutrients. The foliar application of phosphoric acid in combination with the adjuvant LI 700^®^ produced an increase in grain yield when applied at flag leaf emergence but a decrease in grain yield when applied at early tillering. There was high uptake of foliar-applied P regardless of whether it was applied at early tillering or flag leaf emergence. However, translocation of foliar P from the treated leaves to other plant parts was reduced when applied at early tillering compared with flag leaf emergence and is likely due to the high scorch and reduced ability of leaf cells to re-translocate P to other plant parts. The P concentrations in the grain of both control plants and foliar-treated plants (generally <3000 mg kg^-1^) suggest that the plants had marginal P status (Reuter and Robinson, 1997). However, Elliott et al. (1997) found the critical P concentration for grain P at maximum grain yield is between 2,100 and 2,400 mg kg^-1^. Our control plants had grain P concentrations of 2,400 mg kg^-1^, very close to the critical concentration, with all foliar treatments lifting the concentration above this critical value. The addition of phosphoric acid with LI 700^®^ did not increase the P concentration in the grain to an adequate status as defined by the accepted standard of Reuter and Robinson (1997), which implies that further yield response could have been achieved and this may have in part been controlled by the effects of scorch damage.

The degree of scorch was not correlated with yield. However, scorch was very high for all treatments that had drops of fertilizer adhering to the leaves (all fertilizers except the glycerol treatment). It is likely that the scorch score was lower for glycerol only because most of the drops did not adhere to the leaves. The scorch measured in this experiment is unlikely to be a result of the adjuvants themselves, but more likely a combined effect of the low pH and the salt load of the fertilizer solutions, which resulted in scorch scores similar to those described in Peirce et al. (2014) when phosphoric acid was applied at rates equivalent to 1.0 and 2.6 kg P ha^-1^. Although the scorch was not correlated to yield, there is a possibility that the scorch inhibited any potential yield increases that may have resulted from the foliar P application. Reductions in yield with foliar application of P have often been attributed to scorch for a number of different crops (Barel and Black, 1979a; Barel and Black, 1979b; Parker and Boswell, 1980). This could be a direct result of decreased photosynthesis of the plant due to leaf damage (Fageria et al., 2009). We speculate that despite the lack of difference in leaf scorching scores among treatments, the damage caused by the scorching in the LI-700 treatment was less severe and photosynthesis was less impaired in this treatment than with Triton, Agral, and Genapol, hence the larger plant biomass with LI-700 and significantly higher grain yields. The reduction in yield could also be due to differences in general or localised cell death (phytotoxicity) due to the rapid uptake of components of the formulation into the plant cells, as has been documented for herbicides (Zabkiewicz, 2000). As a result of this rapid uptake, the localised death of the leaf cells can in turn reduce the ability of the cells to translocate P and other nutrients from the treated leaves to other plant parts.

The reduced translocation observed when foliar application occurred during tillering may be attributed to the higher phytotoxicity of the formulation at this early growth stage or the reduced ability of the tiller leaves, at their early stage of development, to translocate nutrients out of the leaves. This is consistent with a study by Koontz and Biddulph (1957) which showed that immature bean leaves did not translocate any ^32^P to other plant parts within 24 h compared to fully expanded leaves, which showed rapid translocation. Sargent and Blackman (1962) also found an inverse relationship between the maturity of *Phaseolus vulagris* (French bean) leaves and the rate of 2,4-D with potassium phosphate penetration. It may be that the rapid uptake of foliar applied P by the wheat leaves at the earlier timing resulted in more severe scorch and a reduction in the translocation of P out of the leaves. A younger leaf will also still be a sink for P rather than acting as a source of P for re-translocation. If damage occurs between the timing of foliar application and the leaf changing to a source phase, there may be a reduction of translocation when grown through to maturity.

We show here (as has been shown in other studies (Fernández et al., 2014; Peirce et al., 2014; Peirce et al., 2016) that the adaxial side of wheat leaves, to which we applied the foliar fertilizers, was difficult to wet. Due to the high advancing contact angle and low hysteresis (difference between advancing and receding contact angles), the adaxial leaf side is sometimes classified as superhydrophobic (Lafuma and Quere, 2003). This indicates that water and fertilizers with a surface tension similar to water have difficulty adhering to the leaf surface, resulting in loss of foliar fertilizer and reduced uptake efficiency. In the absence of a surfactant, the contact angle measurements suggest that fertilizer drops were in a Cassie-Baxter state (Cassie and Baxter, 1944) where the drops rested on top of the surface structures (waxes and trichomes). The addition of an adjuvant that contained a surfactant (all adjuvants in this study except glycerol) resulted in a reduction in both the advancing and receding contact angles when compared to water or phosphoric acid alone. In all cases except glycerol, the contact angle reduction resulted in fertilizer drops changing to a Wenzel wetting state (Wenzel, 1936) where the drop penetrated into the surface structure of the leaves resulting in difficulty removing the drop and a receding contact angle of zero. It also means that drops were unlikely to roll off once attached to the leaf.

Previous studies for fertilizers (Koontz and Biddulph, 1957; Fernández et al., 2006; McBeath et al., 2011; Rolando et al., 2014), herbicides, and pesticides (Baker et al., 1992; Gaskin and Holloway, 1992; Stock et al., 1992) have shown that adjuvants can have either a positive or negative effect compared to a control by influencing the uptake of the active ingredient, altering efficacy or affecting yield. For example, Koontz and Biddulph (1957) tested nine different adjuvants in combination with sodium phosphate and found that seven had no effect while two anionic surfactants (Tergitol 7 and Vatsol OTB) decreased the translocation of P in red kidney beans. In contrast, Fisher and Walker (1955) noted a seven-fold increase in the apparent absorption of potassium phosphate with the addition of Triton X-100 by McIntosh apple leaves. However, the studies for fertilizers were rarely performed with wheat and often conducted with plants of unknown leaf wettability (Koontz and Biddulph, 1957). In the case of wettable leaves, the need for a surfactant in the spray solution may not be essential, unlike for wheat. In our study the role of the adjuvant was to reduce the surface tension of the solution and allow it to adhere to the leaf. The adjuvant choice (excluding glycerol) did not change the uptake or translocation of foliar-applied P. This shows that the adjuvant (inclusion of a surfactant or not) needs to be considered in combination with the leaf surface properties (wettable vs. hydrophobic leaves) to maximise foliar fertilizer uptake (Fernandez et al., 2006).

The difference in wettability between the adjuvants is expected as they have different properties. The formulation of the two commercial adjuvants Agral^®^ and LI 700^®^ are somewhat unknown as manufacturers do not disclose exact chemical makeup. Agral^®^ had one active ingredient that is a non-ionic surfactant. It is also made up of 39% non-hazardous (and undisclosed) ingredients. LI 700^®^ is a mixture of propionic acid and soyal phospholipids with multiple modes of action. Due to the emulsion nature of the formulation, homogeneity within the solution was difficult to achieve and resulted in higher variability for contact angles measured. Genapol^®^ X-080 is a non-ionic surfactant, which greatly reduces the surface tension (27 mN m^-1^ at 0.1% (Khayet and Fernández 2012) compared to 72.8 mN m^-1^ for water) of the fertilizer to allow complete wetting of the leaf surface. Triton™ X-100, although also a surfactant, does not reduce the contact angle as drastically as Genapol^®^ X-080. Although there were differences in wettability, the uptake was not affected by the choice of adjuvant with the exception of glycerol and is consistent with the results of Peirce et al. (2016). This may be due to the penetrating ability of the phosphoric acid itself, as evidenced by the high leaf burn that occurs as the P penetrated the leaf surface for the fertilizer treatments that adhered to the leaf.

It is plausible that the grain response measured for the LI 700^®^ treatment occurred due to the humectant properties of the adjuvant compared to the other treatments. The humectant properties arise from the soyal phospholipid part of the LI 700^®^ adjuvant which slows the rate of droplet drying and allows it to stay in solution longer compared to other surfactants. Peirce et al. (2016) also reported a much longer drying time of LI 700^®^ compared to Genapol^®^ X-080 but a similar time to Agral^®^. For this reason, it may be the combination of longer drying time and reduced spread of the droplet (meaning a smaller area of the plant scorched and therefore lower phytotoxicity) which resulted in a positive yield response to phosphoric acid in combination with LI 700^®^. Interestingly, the yield response did not correspond to higher uptake or translocation which might suggest that there was a different scorch effect for this treatment. From this study it is apparent that for phosphoric acid applied to wheat leaves, the foliar P formulation must contain a surfactant, which lowers the surface tension of the formulation, to allow retention of the fertilizer on the leaves. The choice of surfactant is not important for either foliar P uptake or translocation even though different surfactants reduced the contact angle of the fertilizer on the leaves to different degrees. However, it is likely that a formulation which is retained on the leaf (surfactant properties) and stays in solution for longer (humectant properties) will be more likely to produce a positive yield response under controlled environmental conditions.

## Data Availability Statement

The datasets generated for this study are available on request to the corresponding author.

## Author Contributions

CPe contributed to experimental design, completed the experiments, and prepared the manuscript draft. TM contributed to experimental design, analysis, and manuscript preparation for final journal submission. CPr and MM contributed to experimental design, analysis, and manuscript editing.

## Funding

The Grains Research and Development Corporation provided the Grains Industry Research Scholarship and the Fluid Fertilizer Foundation (USA) provided financial support.

## Conflict of Interest

The authors declare that the research was conducted in the absence of any commercial or financial relationships that could be construed as a potential conflict of interest.
